# Army News

**Published:** 1918-09

**Authors:** 


### Army News.

Dr. D. Monroe, of Cameron, Texas, has received word that his son, Captain D. E. Monroe (now acting major and who has been recommended for promotion to that rank), has arrived in France. Major Monroe is in charge of five ambulance companies. There are now three Cameron doctors in France, Major J. S. Denson and Lieut. Van Zandt being the other two.

Perseverance and patriotism found their reward for Dr. Charles Harold Davis of Arlington recently when he received instructions through the county draft board to report to Jefferson Barracks, Mo., for training for the medical reserve corps. Dr. Davis twice tried to enter the corps and was twice rejected for physical disability.

Dr. I. J. Morris, who recently volunteered for service and was given a commission as captain in the Medical Reserve Corps, received orders August 16th to report to Fort Sam Houston, San Antonio for duty.

Dr. J. E. Langston of Dallas was sent to Jefferson Barracks, Mo., recently by the East Dallas Exemption Board. Dr. Langston was subject to limited service under the selective draft, and when a call for two limited service physicians was sent to the East Dallas Board, Dr. Langston was also called into service. Dr. A. R. Super was also sent to Jefferson Barracks by the East Dallas Board.

Captain Isaac Arnold Withers, Medical Reserve Corps, is now stationed at Camp Greenleaf, Fort Oglethorpe, Ga. He was for twenty years a practicing physician in Fort Worth.

Dr. J. A. McConnell of Poolville has volunteered his services and has recently taken examination to enter the medical corps of the United States army.

Dr. John O. McReynolds, a Dallas oculist, has been recommended for appointment to the Medical Reserve Corps, with the rank of major. He will probably be assigned to the aviation service.

Past Assistant Surgean William C. Lyon, Dallas navy recruiting station, has been granted thirty days' leave of absence. Assistant Surgeon Victor B. Riden will assume Dr. Lyon's duties during his absence.

Dr. E. L. Hailey of Celina, Texas, has been made 1st Lieutenant, and ordered to report at Camp Greenleaf, Fort Oglethorpe, Ga., Sept. 1.

Dr. A. P. Baldwin, prominent phyisician of Tyler, received a telegram from the War Department Aug. 21, announcing the death of his son, Lieutenant J. Favre Baldwin, which occured on the firing line in France. Lieutenant Baldwin went to England last fall and after spending several months there was sent to France, where he has been serving in a front line hospital as army surgeon. He was 25 years old and was born and reared in Tyler. He had just entered the practice of medicine after graduating at Tulane University, New Orleons, and was the first native born Tyler citizen to be killed in France.

The son of Dr. Pettit, of Fannin, who has been in France for several months with the American Expeditionary Forces, will soon be discharged from the army. He has been wounded twenty-eight times and recently had all the toes of one foot shot off.

### LAUNCH DRIVE TO ENLIST DOCTORS IN U. S. MEDICAL CORPS.

An active campaign has been launched thoroughout the country to stimulate interest among doctors in the reorganized Volunteer Midical Service Corps. Meetings of the State Committees, Medical Section, Council of National Defense were held in most States on Aug. 22, and another series of meetings for a like purpose has been called for August 29.

The purpose of these meetings is to enlarge the various State executive committees so as to handle an active campaign and to acquaint all State and county representatives with the details of the plans in view. By reason of the fact that the new draft law will include all men up to and including those of 45 years of age, it is anticipated that many doctors, who have not heretofore volunteered their services, may now realize the necessity.

State executive committees will be called to meet on every other Sunday, beginning Sept. 8, and at these meetings the reports of county representatives, together with the consideration of application blanks, will be taken up. County representatives are to be supplied with these blanks and they obligate themselves to see every doctor in their jurisdiction in person or through a trusted representative.

Such doctors will be asked to fill out the application blanks for membership in the Volunteer Medical Service Corps. It is agreed that in no case will a member be called upon until his or her services are needed and in every instance due notice will be given so that important personal business can be taken care of. Instances where doctors refuse to sign the applications will be reported to the Central Governing Board in Washington and the reasons for such refusal cited.

Dr. E. B. Clements has received information from his son, Capt. P. C. Clements, of the Medical Corps, that he has arrived hale and hearty in France.

First Lieutenant James T. Colwick, a Dallas physician, second in charge of surgery at Love Field, was commissioned captain on Aug. 24.

Dr. Arthur M. Freels, a physician of Denison, has been commissioned a captain in the medical department, and left Denison Aug. 24 for Fort Sam Houston to take up his new duties.

Dr. Harvey Oliver of Fort Worth, has been appointed captain in the reserve medical corps, according to word received from Washington.

Dr. L. N. Conally, who left Beeville recently with a draft contingent for Camp Travis, has been moved and is now at Camp Merritt, N. J.

Dr. A. C. Rogers of Waxahachie has been commissioned a First Lieutenant in the medical reserve corps. He left September 1 for Fort Oglethorpe, Ga.

Six Dallas doctors will leave soon for France. They are: Drs. W. M. Young, W. D. Jones, A. R. Super, U. P. Hackney, A. M. Spurgeon and J. M. Black.

